# Supplementary material for: Comparative multiomics analysis of cell physiological state after culture in a basket bioreactor
Source: Sci Rep. 2022 Nov 23;12:20161. doi: 10.1038/s41598-022-24687-4 (PMC9686226; doi:10.1038/s41598-022-24687-4)

66041.1:168400522:168401075:-@NW_023666041.1:168400167:168401075:-@NW_023666041.1:168400167:168

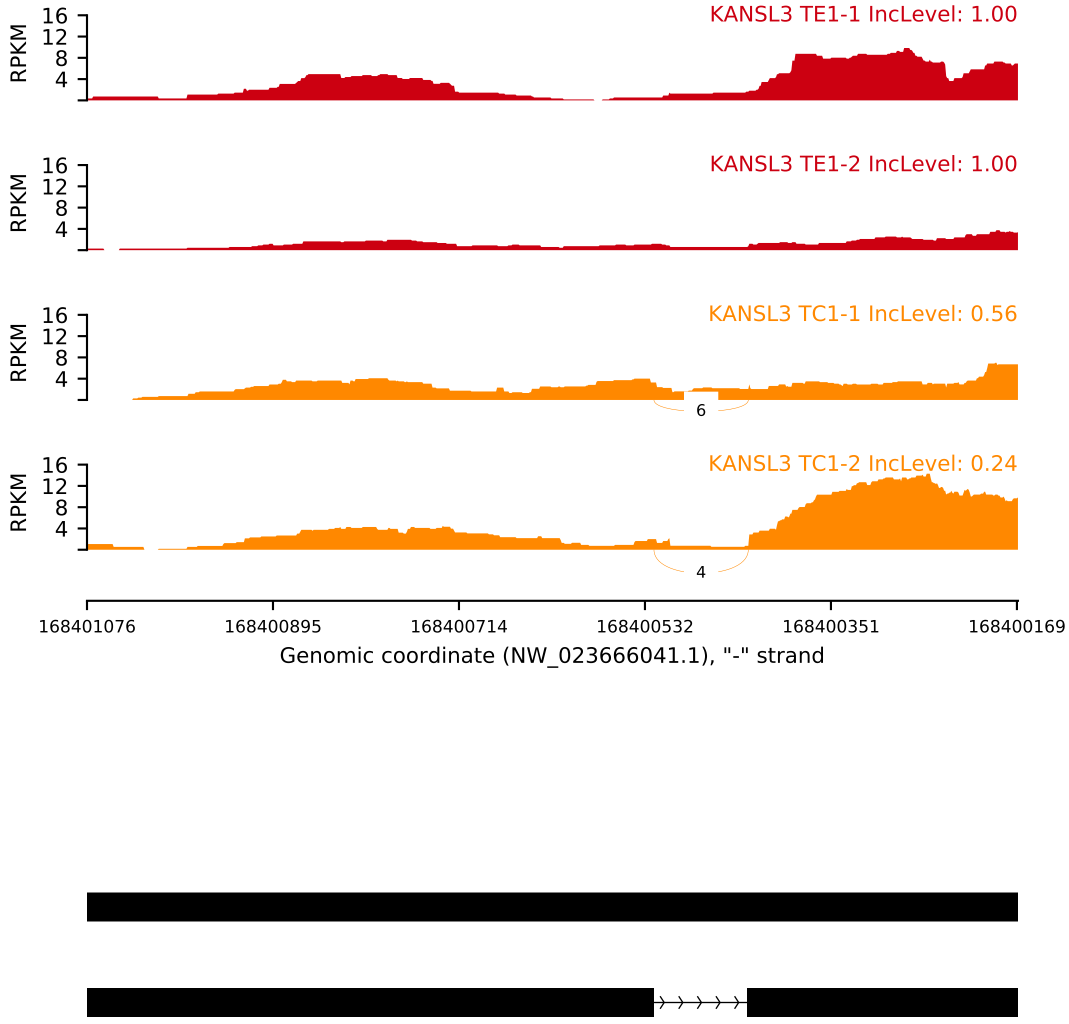

Supplement: Supplementary file 1 — Supplementary Information 1. [file 41598_2022_24687_MOESM1_ESM.zip › raw data/Transcriptomic raw data/7.AS/2.dasplot/TE1vsTC1/RI/8_KANSL3_NW_023666041.1_168400522_168401075_-@NW_023666041.1_168400167_168401075_-@NW_023666041.1_168400167_168400431_-.pdf]
